# 4007 Medroxyprogesterone Upregulates the Glucocorticoid Receptor in Female Long Evans Rats

**DOI:** 10.1017/cts.2020.80

**Published:** 2020-07-29

**Authors:** Margaret Zimmerman, Benard Ogola, Sarah Lindsey

**Affiliations:** 1 Tulane University School of Medicine- LA CaTS; 2 Tulane University, New Orleans, LA, USA

## Abstract

OBJECTIVES/GOALS: Estrogen monotherapy in postmenopausal women can reduce kidney function, while dual therapy combining estrogen with a progestin improves renal health. Using the female Long Evans rat as a novel animal model of postmenopausal cardiovascular disease, we found similar results where estrogen worsens renal health while co-administration of medroxyprogesterone acetate (MPA) was protective. MPA cross-activates glucocorticoid receptors (GR), which are targeted clinically for their anti-inflammatory actions. Therefore, our goal was to determine if estrogen monotherapy induces renal damage by increasing inflammation, while dual therapy with MPA opposes inflammation by cross-activating GR. METHODS/STUDY POPULATION: Female Long Evans rats underwent OVX at 11 months of age and received a subcutaneous implant containing E2, E2+MPA or vehicle for 40 days. RESULTS/ANTICIPATED RESULTS: Coadministration of MPA prevented the E2-induced increase in proteinuria (Veh: 0.27 ± 0.07; E2: 3.53 ± 1.16; E2 + MPA: 1.20 ± 0.58 mg/mg creatinine; P = 0.03) and decline in glomerular filtration rate (Veh: 0.51 ± 0.02; E2: 0.24 ± 0.05; E2+MPA: 0.39 ± 0.05 ml/min; P < 0.01). Co-administration of MPA significantly increased renal GR transcript levels compared with E2 alone (Veh: 0.96 ± 0.02; E2: 0.94 ± 0.10; E2+MPA: 1.24 ± 0.04 fold change; P < 0.01). Inflammatory marker COX 2 renal transcript levels were significantly reduced by a similar degree in both mono and dual therapies compared with vehicle (Veh: 1.07 ± 0.06; E2: 0.81 ± 0.04; E2+MPA: 0.81 ± 0.04 fold change; P < 0.01). Neither TNF-alpha and IL-6 mRNA nor urinary beta-microglobulin levels (Veh: 1.71 ± 0.31; E2: 2.88 ± 0.78; E2+MPA: 3.07 ± 1.15 mg/day; ns) were altered. DISCUSSION/SIGNIFICANCE OF IMPACT: Our results show that the effect of E2 on renal pro-inflammatory markers was not altered by the addition of MPA despite the significant increase in renal GR levels. Therefore, the renoprotective effects of MPA in midlife hormone therapy may be independent of renal GR-mediated changes in the immune profile.

